# Remarkably similar CTLA-4 binding properties of therapeutic ipilimumab and tremelimumab antibodies

**DOI:** 10.18632/oncotarget.18004

**Published:** 2017-05-19

**Authors:** Mengnan He, Yan Chai, Jianxun Qi, Catherine W.H. Zhang, Zhou Tong, Yi Shi, Jinghua Yan, Shuguang Tan, George F. Gao

**Affiliations:** ^1^ CAS Key Laboratory of Pathogenic Microbiology and Immunology, Institute of Microbiology, Chinese Academy of Sciences, Beijing 100101, China; ^2^ University of Chinese Academy of Sciences, Beijing 100049, China; ^3^ ImmuFuCell Biotechnology Co., Ltd., Beijing 100102, China; ^4^ CAS Key Laboratory of Microbial Physiological and Metabolic Engineering, Institute of Microbiology, Chinese Academy of Sciences, Beijing 100101, China

**Keywords:** ipilimumab, CTLA-4, complex structure, tremelimumab

## Abstract

Monoclonal antibody based immune checkpoint blockade therapies have achieved clinical successes in management of malignant tumors. As the first monoclonal antibody targeting immune checkpoint molecules entered into clinics, the molecular basis of ipilimumab-based anti-CTLA-4 blockade has not yet been fully understood. In the present study, we report the complex structure of ipilimumab and CTLA-4. The complex structure showed similar contributions from VH and VL of ipilimumab in binding to CTLA-4 front β-sheet strands. The blockade mechanism of ipilimumab is that the strands of CTLA-4 contributing to the binding to B7-1 or B7-2 were occupied by ipilimumab and thereafter prevents the binding of B7-1 or B7-2 to CTLA-4. Though ipilimumab binds to the same epitope with tremelimumab on CTLA-4 with similar binding affinity, the higher dissociation rate of ipilimumab may indicate the dynamic binding to CTLA-4, which may affect its pharmacokinetics. The molecular basis of ipilimumab-based anti-CTLA-4 blockade and comparative study of the binding characteristics of ipilimumab and tremelimumab would shed light for the discovery of small molecular inhibitors and structure-based monoclonal antibody optimization or new biologics.

## INTRODUCTION

The approval of anti-CTLA-4 monoclonal antibody (MAb), ipilimumab (MDX-010, Yervoy) from Bristol-Myers Squibb (New York, US), by US Food and Drug Administration (FDA) in 2011 has initiated a new era for tumor immunotherapy. The “two signal” model of naïve T cell activation involves the recognition of T cell receptor (TCR) and peptide major histocompatibility complex (pMHC) as the first signal, and the interaction between co-stimulators and their ligands, such as CD28 and B7-1, as the second signal [1–4]. CTLA-4 is a member of CD28-B7 immunoglobulin superfamily of immune regulatory molecules which acts as a negative regulator of T cell activation, especially CD28-dependent T cell responses [5]. The activation or exhaustion of T cells depends strongly on the co-stimulatory and co-inhibitory signaling pathways, therefore the co-stimulatory and co-inhibitory molecules are also termed as “immune checkpoint” molecules [6–8]. CTLA-4 functions to inhibit T cell activity via the binding to common ligands with CD28, the B7-1 (CD80) or B7-2 (CD86), with significantly higher binding affinity than that with CD28 [9]. The complex structures of CTLA-4 and B7-1 or B7-2 have been determined, showing a similar binding mode to these two ligands [10, 11]. It has been found that the blockade of CTLA-4 signaling with MAbs would enhance the antitumor immunity in mouse model [12]. Thereafter, investigations of checkpoint blockade immunotherapy with MAbs that could block PD-1, PD-L1, etc. have been extensively studied from bench to bedside. The anti-CTLA-4 ipilimumab has been used for the treatment of melanoma in clinics and also been extensively studied in dealing with multiple tumors in either monotherapy or combination with other checkpoint blockade therapeutics [6, 13, 14]. Multiple clinical studies revealed that monotherapy of ipilimumab in metastasis melanoma patients showed an overall responsive rate (ORR) of 10-20% while co-administration with other checkpoint therapeutics could substantially raise the ORR [13, 15]. However, the molecular basis of ipilimumab-based anti-CTLA-4 blockade for tumor immunotherapy has not yet been fully revealed, which has restricted our understanding of this MAb. Moreover, there is another anti-CTLA-4 MAb, tremelimumab (CP-675,206) from Medimmune, AstraZeneca, in multiple phase III clinical trials (NCT02369874, NCT02453282, etc.), whose CTLA-4-binding basis has just been revealed lately [16].

It has been hypothesized that anti-CTLA-4 for tumor immunotherapy is achieved by releasing brakes on both regulatory T cells (Tregs) and conventional T cells. The administration with anti-CTLA-4 MAbs may involve two activities, intervention of CTLA-4/B7-1 (or B7-2) interaction via the binding of the MAbs; and depletion of targeted cells, e.g. immunosuppresive Tregs, via Fc mediated antibody-dependent cell-mediated cytotoxicity (ADCC) and complement dependent cytotoxicity (CDC). Ipilimumab is an IgG1 MAb while tremelimumab is IgG2 with different ADCC and CDC activities [17]. Clinical performances of these two MAbs exhibit substantial differences that administration of tremelimumab showed no significant survival advantage over standard-of-care chemotherapy [18]. Whether the differences of anti-tumor efficacy is dependent on the intervention of CTLA-4/B7-1 (or B7-2) interaction or Fc-mediated ADCC/CDC activity remains unknown. Recently, the structural basis of checkpoint blockade MAbs has been revealed for PD-1 targeting nivolumab and pembrolizumab, PD-L1 targeting avelumab and BMS-936559, and CTLA-4 targeting tremelimumab [8, 16, 19–21]. The determination of the molecular basis of ipilimumab based anti-CTLA-4 activity and comparison of the binding characteristics between ipilimumab and tremelimumab are of significance for our understanding of anti-CTLA-4 immunotherapy and the design of next-generation anti-CTLA-4 therapeutics.

In the present study, the molecular basis for ipilimumab based anti-CTLA-4 is revealed through the determination of ipilimumab/CTLA-4 complex structure. Comparative study with tremelimumab is also conducted to elucidate the structural basis and binding characteristics of these two anti-CTLA-4 MAbs which are either clinically used or under multiple Phase III trials.

## RESULTS

### Overall structure of ipilimumab-scFv/CTLA-4 complex

**Figure 1:**
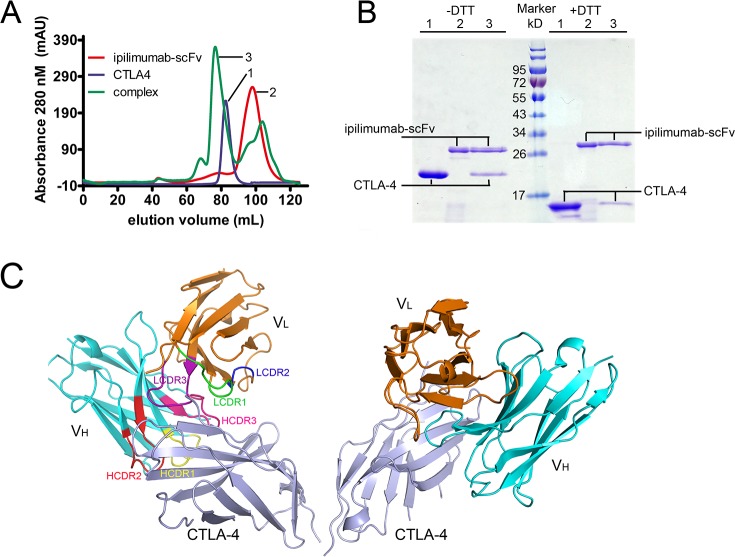
Overall structure of ipilimumab-scFv/CTLA-4 complex **(A)** Gel filtration profiles of the CTLA-4, ipilimumab-scFv and ipilimumab-scFv/CTLA-4 complex were analyzed by size-exclusion chromatography as indicated. The X axis represents the elution volume of each protein. The ipilimumab-scFv/CTLA-4 complex can survive well on the gel filtration column. **(B)** SDS-PAGE assay confirmation of the ipilimumab-scFv/CTLA-4 complex protein. The CTLA-4 forms a homodimer through cystine mediated disulfide bond, as indicated in line 1 and line 3 without DTT. The presence of ipilimumab-scFv band and CTLA-4 band in line 3 supports the formation of ipilimumab-scFv/CTLA-4 complex. **(C)** Overall structure of the ipilimumab-scFv/CTLA-4 complex. CTLA-4 is shown as cartoon representations in light blue, and the heavy (VH) and light chains (VL) of scFv are shown in cyan and orange, respectively. The CDR1, CDR2 and CDR3 loops of VH are colored in yellow, red and hot pink, respectively. The CDR1, CDR2 and CDR3 loops of VL are colored in green, blue and purple, respectively.

**Figure 2:**
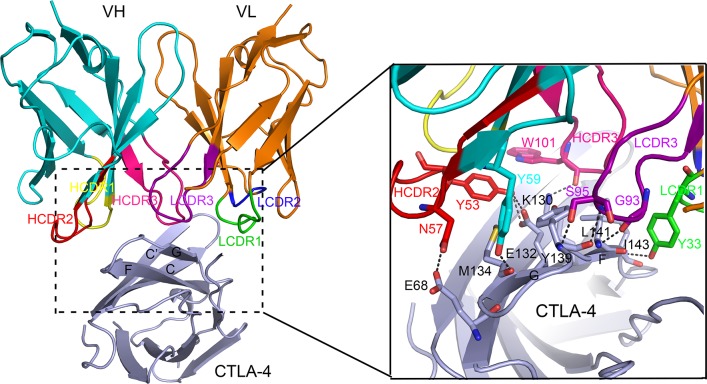
Detailed interactions within the interface of ipilimumab-scFv/CTLA-4 complex Residues involved in the hydrogen bond interactions are shown as sticks and labeled. Hydrogen bonds are shown as dash lines.

We expressed the single chain Fv fragment (scFv) of ipilimumab and the extracellular domain of CTLA-4 as inclusion bodies in *E. coli*. Soluble proteins were obtained through *in vitro* refolding method and ipilimumab-scFv/CTLA-4 complex protein could be obtained after co-incubation (Figure 1A, 1B). Crystals were then screened with ipilimumab-scFv/CTLA-4 complex protein and well-diffractable crystals were obtained (see more details in materials and methods). The complex structure of the CTLA-4 and ipilimumab-scFv was determined by molecular replacement (PDB: 1I8L and 3F12 as search models) at a resolution of 3.3 Å (Table 1). In this complex structure, CTLA-4 presented as a homodimer as previously reported and the binding by ipilimumab didn’t affect the dimer formation (Figure 1C) [11]. CTLA-4 is an immunoglobulin molecule constituted by two β-sheet faces, the front A’GFCC’ β-sheet face and the back ABED β-sheet face. Overall, ipilimumab utilized both of its heavy chain (VH) and light chain (VL) to bind to CTLA-4 with a buried surface of 1708.9 Å^2^ (Figure 2). Specifically, HCDR2 and HCDR3 of VH domain and LCDR3 of VL provided predominant contacts to CTLA-4 with partial contributions from LCDR1 (Figure 2). The binding of ipilimumab on CTLA-4 mainly located on the front β-sheet face constituted by C, G and F strands with multiple hydrogen bonds to E68 on C strand, K130, E132 and M134 on F strand, and Y139, L141 and I143 on G strand of CTLA-4 (Figure 2, Table 2). Within this interface, VH and VL contributed equally with similar numbers of hydrogen bonds to CTLA-4, i.e. five hydrogen bonds for VH vs four hydrogen bonds for VL, respectively. Taken together, the complex structure revealed that ipilimumab binds to CTLA-4 front β-sheet face with similar contributions from VH and VL domains.

**Table 1:** Data collection and refinement statistics

Space group	P222
Wavelength (Å)s	0.97889
Unit cell dimensions	
a, b, c (Å)	91.96, 114.24, 150.12
α, β, γ (°)	90.00, 90.00, 90.00
Resolution (Å)	50.00–3.20 (3.31–3.20)^1^
Observed reflections	26870
Completeness (%)	99.9 (100.0)
Redundancy	7.0 (7.1)
R_merge_ (%)	21.7 (168.8)
I/σ	9.6 (1.3)
Refinement	
R_work_ / R_free_(%)	22.2/26.4
No. atoms	
Protein	10424
Water	0
*B*-factors	
Protein	69.3
Water	-
r.m.s. deviation	
Bond lengths (Å)	0.0036
Bond angles (°)	0.78
Ramachandran plot	
Favored (%)	95.5
Allowed (%)	4.5
Outliers (%)	0.0

^1^ Values in parentheses are given for the highest resolution shell.

**Table 2:** Interactions between ipilimumab and CTLA-4

	Antibody	CTLA-4	Contacts	Total contacts
H chain (VH)	W101	L74, V81, T82, E83, K130 (1) ^2^, I143	2, 6, 2, 13, 8, 1	135 ^1^
	L102	L74, V81, L141, I143	1, 1, 1, 4	
	G103	L141	2	
	S31	E83	4	
	T33	E132	1	
	F50	Y139	3	
	S52	M134	2	
	Y53	R70, K130, E132 (2), M134	21, 2, 9, 2	
	N57	E68 (1), M134, Y135	7, 6, 4	
	Y59	M134 (1), Y135, P136, P137, Y139	8, 4, 2, 14, 5	
L chain (VL)	Q27	K36	2	80
	Y33	L141, G142, I143 (1), M38	11, 5, 7, 1	
	Y50	S79	1	
	Y92	L141	4	
	G93	Y139, Y140, L141 (1)	1, 2, 10	
	S94	Y139, Y140	4, 2	
	S95	P138, Y139 (2)	7, 14	
	W97	Y139	9	

^1^ Numbers represent the number of atom-to-atom contacts between the antibody residues and the CTLA-4 residues, which were analyzed by the contact program in CCP4 suite (the distance cutoff is 4.5 Å).

^2^ Numbers in the parentheses represent the number of hydrogen bonds between the antibody residues and the CTLA-4 residues, which were analyzed by the contact program in CCP4 suite (the distance cutoff is 3.5 Å).

### Structural basis of ipilimumab based CTLA-4/B7-1 blockade

**Figure 3:**
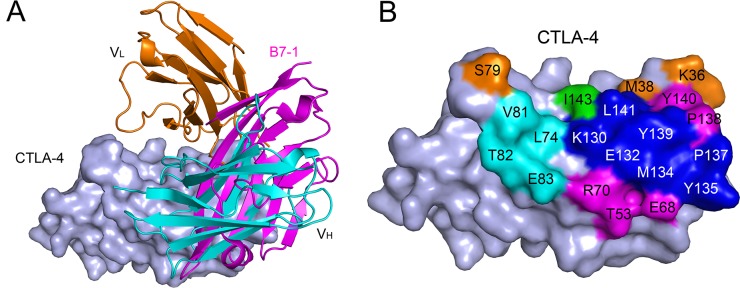
Competitive binding of ipilimumab-scFv and B7-1 with CTLA-4 **(A)** Superposition of the ipilimumab-scFv/CTLA-4 complex structure with CTLA-4/B7-1 complex structure (PDB: 1I8L). CTLA-4 is shown as surface diagram in light blue, B7-1 in pink, ipilimumab-scFv VH in cyan, VL in orange, respectively. **(B)** Binding surface of CTLA-4 by B7-1 or ipilimumab. The binding residues by B7-1 on CTLA-4 are colored in pink, whereas residues contacted by the ipilimumab-scFv VH or VL are colored in cyan or orange, respectively, while residues contact with both VH and VL are colored in green, and the overlapping residues bound by both B7-1 and ipilimumab are colored in blue.

Structural basis of CTLA-4/B7-1 blockade was analyzed by structural superposition of the human CTLA-4/B7-1 complex (PDB: 1I8L) and the ipilimumab-scFv/CTLA-4 complex. The results revealed that the binding of ipilimumab and B7-1 to CTLA-4 has stereo clash to each other (Figure 3A). The binding region on CTLA-4 by ipilimumab was overlapped with that by B7-1 (Figure 3B). The interaction of CTLA-4 and B7-1 involved both of the front β-sheet faces of their Ig domain [11]. The overlapped binding surface on CTLA-4 by B7-1 and ipilimumab was also predominantly located on both F strand (K130, E132, M134 and Y135) and G strand (P137, Y139 and L141) of CTLA-4, the very region occupied by VH and VL of ipilimumab (Figure 3B). These results indicated that the blockade mechanism of ipilimumab was that the VH and VL of ipilimumab binds to the same region on CTLA-4 by B7-1 or B7-2 and thereafter prevents the binding to CTLA-4.

### Ipilimumab and tremelimumab bind to the same region on CTLA-4 with distinct paratope

**Figure 4:**
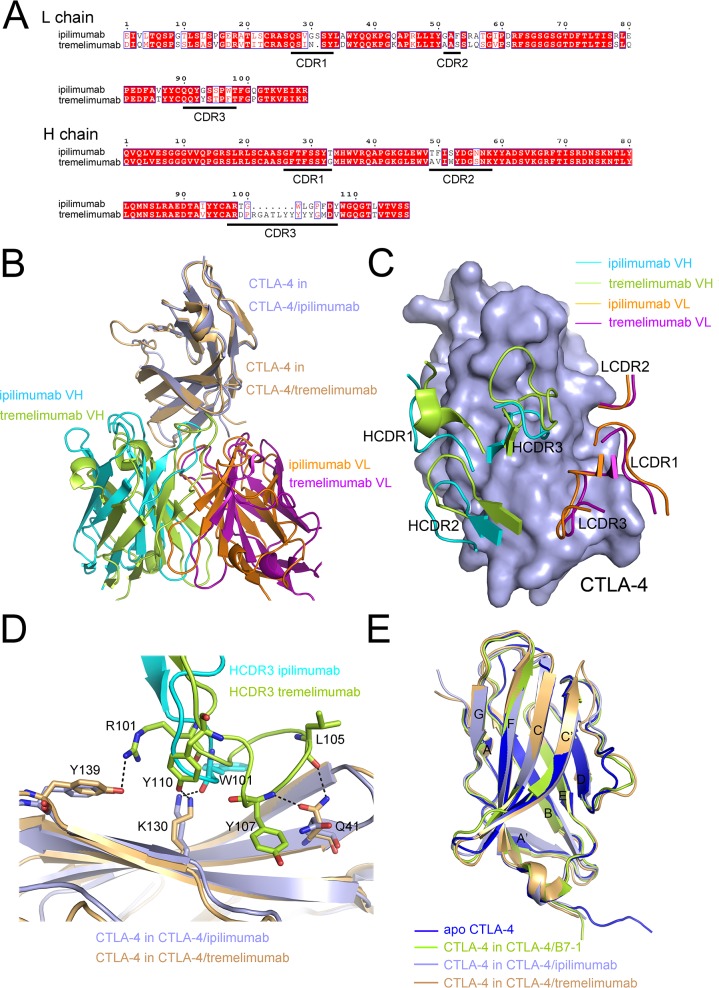
Similar epitope and distinct binding mode of ipilimumab and tremelimumab **(A)** Sequence alignment of the VH and VL of ipilimumab and tremelimumab with CDRs indicated as underlined. **(B)** Superposition of ipilimumab-scFv/CTLA-4 complex and tremelimumab-Fab/CTLA-4 complex. CTLA-4 is shown in light blue. The VH of ipilimumab colored in cyan, VH of tremelimumab colored in limon, VL of ipilimumab colored in orange and VL of tremelimumab colored in purple, respectively. **(C)** Comparative binding of the CDR loops in ipilimumab and tremelimumab to CTLA-4 with VH of ipilimumab colored in cyan, VH of tremelimumab colored in limon, VL of ipilimumab colored in orange and VL of tremelimumab colored in purple, respectively. **(D)** Detailed interactions of HCDR3 of both ipilimumab and tremelimumab with CTLA-4. Residues involved in the hydrogen bond interactions are shown as sticks and labeled. Hydrogen bonds are shown as dash lines. HCDR3 of ipilimumab is colored in cyan, HCDR3 of tremelimumab in limon, the CTLA-4 in CTLA-4/ipilimumab complex in light blue, and CTLA-4 in CTLA-4/tremelimumab complex in light orange, respectively. **(E)** Comparison of the conformational changes of CTLA-4s extracted from ipilimumab-scFv/CTLA-4 complex (light blue), B7-1/CTLA-4 complex (limon), tremelimumab-Fab/CTLA-4 (hot pink) and free CTLA-4 (blue). β-sheets were labeled as indicated.

Structural basis of the two clinically available anti-CTLA-4 MAbs, the ipilimumab and tremelimumab, was further compared to elucidate the molecular mechanism of the binding with CTLA-4 and intervention of CTLA-4/B7-1 interaction. Tremelimumab and ipilimumab use similar VH framework regions, IGHV3-33 of tremelimumab and IGHV3-30 of ipilimumab, while the VL framework regions were completely different from each other, IGKV1-39 of tremelimumab and IGKV3-20 of ipilimumab (Figure 4A). Superposition of tremelimumab/CTLA-4 (PDB: 5GGV) and ipilimumab/CTLA-4 complex structures revealed a remarkably similar binding mode of these two MAbs (Figure 4B). The CDRs of the VL of ipilimumab and tremelimumab showed no substantial differences compared with each other (Figure 4C). On the other hand, CDRs of the VH of ipilimumab revealed completely different conformation with that of tremelimumab (Figure 4C). Especially, the HCDR3 was completely different from each other that the HCDR3 in tremelimumab was much longer, 18 aa for tremelimumab HCDR3 vs 11 aa for ipilimumab HCDR3. Detailed interactions involving HCDR3 of both MAbs to CTLA-4 revealed that more hydrogen bonds were contributed from HCDR3 of tremelimumab than that of ipilimumab, 4 hydrogen bonds in tremelimumab HCDR3 vs 1 hydrogen bond in ipilimumab HCDR3, indicating the substantial binding differences of these two MAbs (Figure 4D). Superposition of CTLA-4 molecules extracted from complexes of ipilimumab-scFv/CTLA-4, tremelimumab/CTLA-4 or CTLA-4/B7-1 (PDB: 1I8L) and free CTLA-4 (PDB: 3OSK) revealed that CTLA-4 showed no substantial differences among these complexes except for minor variations in some of the loops that connect the strands (Figure 4E). The variations in the loops may be explained by the flexibility of these loops while none of these loops were participated in the interaction with MAbs or B7-1. These analyses revealed that ipilimumab and tremelimumab bind to the same region on CTLA-4 with distinct binding mode, mainly contributed by the VH of these two MAbs.

### Distinct binding kinetics of the ipilimumab and tremelimumab

**Figure 5:**
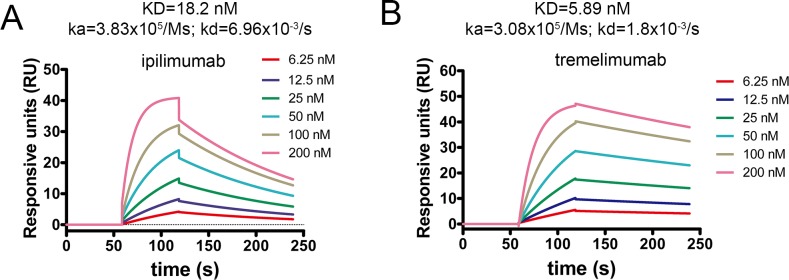
Binding profiles of ipilimumab-scFv/CTLA-4 and tremelimumab-scFv/CTLA-4 measured by SPR CTLA-4 was immobilized on the chip while serially diluted **(A)** ipilimumab-scFv or **(B)** tremelimumab-scFv with concentrations ranging from 6.25 nM to 200 nM were then flowed through the chip and the response units were measured.

Binding profiles of these two antibodies with CTLA-4 were further investigated using surface plasmon resonance (SPR) analysis. The binding affinity of ipilimumab to CTLA-4 (KD=18.2 nM) was much higher than that of B7-1 as previously reported (KD=0.42 μM), which enables the exploitative binding to CTLA-4 (Figure 5A) [9]. The binding affinity to CTLA-4 showed no substantial differences between ipilimumab and tremelimumab (KD=5.89 nM) (Figure 5B). However, the dissociation rate constant (kd) of ipilimumab (kd = 6.96×10^−3^/s) was much higher than that of tremelimumab (kd = 1.8×10^−3^/s) while the association rate constant (ka) for ipilimumab (ka = 3.83×10^5^/Ms) and tremelimumab (ka = 3.08×10^5^/Ms) showed no substantial differences between each other. These results indicated that the binding of ipilimumab with CTLA-4 was less stable than that of tremelimumab. However, whether these differences would affect the administration of these MAbs clinically yet needs further investigations.

## DISCUSSION

In the present study, we reported the structural basis of the first clinically applied anti-CTLA-4 MAb, the ipilimumab. Though Fc mediated ADCC or CDC activity was important for the depletion of CTLA-4 high Tregs, the blockade of CTLA-4/B7-1 (B7-2) to restore conventional T cell reactivity was also critical for its anti-tumor efficacy [7, 12, 13, 22]. The determination of ipilimumab/CTLA-4 complex structure has provided useful information for our understanding of how this MAb work to blockade the interaction between CTLA-4 and B7-1 (or B7-2). The recent report of nonpeptidic chemical inhibitors targeting PD-L1 to block the interaction of PD-1/PD-L1 suggests that there are “hot spots” on checkpoint molecules for the design of small molecular inhibitors [23]. The small molecular therapeutics, such as high-affinity PD-1, has been proven to be more efficient in tumor penetration than MAbs, indicating favorable pharmacology of small therapeutics for enhanced cancer immunotherapy [24, 25]. Thus, the complex structure presented here would be helpful for the design of small molecular inhibitors for CTLA-4 to improve anti-tumor efficacy.

The present study reveals the molecular basis of ipilimumab based anti-CTLA-4 activity. The binding mode of the ipilimumab via both H and L chains to the F and G strands of CTLA-4 was different to MAbs based PD-1/PD-L1 blockade. Previous structural analysis of the PD-1/PD-L1 blockade MAbs showed a “loop”-dominating binding to these immune checkpoint molecules, e.g. C’D loop of PD-1 by pembrolizumab and N-terminal loop of PD-1 for nivolumab, FG loop of PD-L1 for avelumab [16, 19, 26]. Together with the apo CTLA-4 and CTLA-4s in complex with tremelimumab or B7-1, all the loops of CTLA-4 were not involved in the interaction with the counterparts. Therefore, unlike the “loop”-dominated interaction of PD-1 or PD-L1 with MAbs, the loops of CTLA-4 were left untouched in the interaction with MAbs, while major contacts were provided by the strands of the core domain of CTLA-4. Moreover, the region on PD-1 or PD-L1 contributed major interaction with these MAbs was quite different from that for competitive intervention. Here, the complex structure of ipilimumab/CTLA-4 reveals that the region on CTLA-4 contributed major interaction with ipilimumab is exactly the region deprived by ipilimumab for competitive intervention.

Ipilimumab and tremelimumab are the only two anti-CTLA-4 MAbs that were under clinical investigations in dealing with multiples tumors. Administration of ipilimumab has achieved significant clinical benefits in management of advanced melanoma from multiple trials [14, 15, 22, 27]. On the other hand, treatment with tremelimumab failed to show a statistically significant survival advantage in advanced melanoma patients [18]. However, treatment with tremelimumab has shown encouraging clinical activity in dealing with multiple tumors and was thus still under multiple clinical trials in combination therapy [28, 29]. The ipilimumab is an IgG1 isotype while tremelimumab is IgG2. Clarification of whether the differences of the clinical performances of these two MAbs is due to the V segment dependent CTLA-4 targeting or Fc mediated ADCC/CDC activity is critical for the understanding of anti-CTLA-4 tumor immunotherapy. Previous studies revealed that ipilimumab can engage *ex vivo* FcγRIIIA (CD16)-expressing, nonclassical monocytes resulting in ADCC-mediated lysis of Tregs [30]. Though there is no direct report about the ADCC/CDC activity of tremelimumab, the IgG2 subclass of tremelimumab indicates that it would less likely induce ADCC/CDC activity [17]. The results from the present study showed that ipilimumab and tremelimumab bind to the same region on CTLA-4 with similar binding affinity but distinct paratope. The similarities of the binding sites of these two MAb indicate a hot-spot for anti-CTLA-4 therapeutic MAbs interaction and may serve as a target for future small molecular biologics development. The higher dissociation rate of ipilimumab than that of tremelimumab indicates the dynamic binding of ipilimumab and may affect its pharmacokinetics. However, whether the differences of the binding kinetics of these two MAbs would affect the anti-tumor efficacy yet need further investigations. We propose that the differences of the clinical performances of these two MAbs may mainly derived from their IgG isotypes.

Taken together, the findings in the present study elucidated the molecular basis of ipilimumab-based anti-CTLA-4 immunotherapy. Though the binding epitope of ipilimumab resembles that of tremelimumab with similar binding affinity, the substantial differences of the binding paratope of these two MAbs and the higher dissociation rate of ipilimumab indicate the distinct pharmacokinetics. More importantly, the similar binding sites of ipilimumab and tremelimumab may serve as hot-spot for anti-CTLA-4 MAbs development and opens the door to the chance for discovery of small molecular inhibitors and structure-based MAb optimization.

## MATERIALS AND METHODS

### Protein preparation

The DNA encoding the ectodomain of human CTLA-4 (aa 31-161) (Uniprot: P16410-1) was cloned into pET-21a vector (Invitrogen). The DNA sequence for the scFv fragment of ipilimumab and tremelimumab were constructed as VL-GG(GGSGG)_3_GG-VH and cloned into the pET21a expression vector (Invitrogen) (Supplementary Table 1). All plasmids were transformed into *E. coli* BL21 (DE3) and expressed as inclusion bodies. The cells were grown at 37°C in LB medium supplemented with 50 μg/ml ampicillin until OD600 reached 0.6–1.0, and the protein expression was induced with 1 mM Isopropyl β-D-1-thiogalactopyranoside (IPTG) and incubated for 5 h at 37°C. The cells were harvested by centrifugation, re-suspended in phosphate buffer saline (PBS) and lysed by high-pressure homogenization (JNBIO, China). Inclusion bodies were recovered by centrifugation (12, 000 rpm/min for 20 min at 4°C) and solubilized in 50 mM Tris, 100 mM NaCl, 10 mM EDTA, 6 M guanidine hydrochloride (Gua-HCl), 10 mM dithiothreitol (DTT), 10% glycerol, pH 8.0 by stirring overnight. After removing undissolved protein by centrifugation (12, 000 rpm/min for 10 min at 4°C), the solubilized proteins were diluted into refolding buffer (100 mM Tris, 400 mM L-Arginine-HCl, 2 mM EDTA, 5 mM GSH, 0.5 mM GSSG, pH 8.0) by stirring for 8-10 h [19]. Subsequently, the refolded proteins were concentrated using an Amicon 400 concntrator with 10 kDa cut-off membrane and then adjusted to 20 mM Tris, 150 mM NaCl, 5% glycerol, pH8.0. The proteins were purified in an ÄKTA Pure (GE Healthcare Life Sciences) by gel filtration chromatography using a HiLoad 16/600 Superdex™ 200pg column (GE Healthcare Life Sciences). The protein qualities were evaluated by reduced and nonreduced 15% SDS–PAGE gel and stained with Coomassie blue.

The ipilimumab-scFv/CTLA-4 complex was formed by incubating CTLA-4 and ipilimumab in 1:2 molar ratio at 4°C for 2 h. The sample was then purified using a HiLoad 16/600 Superdex™ 200pg column (GE Healthcare Life Sciences). The pooled proteins were analyzed on 15% SDS-PAGE gel and stained with Coomassie blue.

### Crystal screening, data collection and structural determination

The purified ipilimumab-scFv/CTLA-4 complex was concentrated to 10 mg/mL for crystallization. Crystals were generated at 18°C using sitting-drop vapour-diffusion method by mixing 1 μL protein solution and 1 μL reservoir solution. Crystals of ipilimumab-scFv/CTLA-4 complex grew in 100 mM BICINE, 10% PEG 20000, 2% 1,4-Dioxane, pH 9.0.

For data collection, all crystals were cryo-protected by briefly soaking in the mixture of 2.5 μL crystallization buffer and 1 μL 20% (v/v) glycerol before flash-cooling in liquid nitrogen. Diffraction data were collected at Shanghai Synchrotron Radiation Facility (SSRF) BL17U. All the datasets were processed with HKL2000 software [31]. The structures of ipilimumab-scFv and CTLA-4 were determined by the molecular replacement method using Phaser with previously reported antibody (PDB: 3F12) and CTLA-4 protein structure (PDB: 1I8L) as the search model [32]. The atomic models were completed with Coot [33] and refined with Phenix [34]. The stereochemical qualities of the final model were assessed with MolProbity. All structure figures were prepared with Pymol (http://www.pymol.org). Coordinates and structure factor of the structure reported here have been deposited into the Protein Data Bank with PDB Code: 5XJ3.

### SPR analysis

SPR measurements were done at room temperature using a BIAcore T100^R^ system with CM5 chips (GE Healthcare). For all measurements, an HBS-EP buffer consisting of 150 mM NaCl, 10 mM HEPES, pH 7.4 and 0.005% (v/v) Tween-20 was used as running buffer, and all proteins were exchanged into this buffer in advance through gel filtration. The blank channel of the chip served as the negative control. To detect different antibody (ipilimumab or tremelimumab) binding to CTLA-4, CTLA-4 was immobilized on the chip at a concentration of 5 μg/ml. Gradient concentrations of ipilimumab and tremelimumab (6.25 nM, 12.5 nM, 25 nM, 50 nM, 100 nM and 200 nM) were then flowed over the chip surface. After each cycle, the sensor surface was regenerated with Glycine, pH 1.5. The binding kinetics were all analysed with the software of BIA evaluation^R^ Version 4.1 using a 1:1 Langmuir-binding model.

## SUPPLEMENTARY TABLE



## Abbreviations

Mab: monoclonal antibody
TCR: T cell receptor
pMHC: peptide major histocompatibility complex
ORR: overall responsive rate
Tregs: regulatory T cells
ADCC: antibody-dependent cell-mediated cytotoxicity
CDC: complement dependent cytotoxicity
scFv: single chain Fv fragment
VH: heavy chain
VL: light chain
SPR: surface plasmon resonance
kd: dissociation rate constant
ka: association rate constant
