# Supplementary material for: Remarkably similar CTLA-4 binding properties of therapeutic ipilimumab and tremelimumab antibodies
Source: Oncotarget. 2017 May 19;8(40):67129–39. doi: 10.18632/oncotarget.18004 (PMC5620161; doi:10.18632/oncotarget.18004)
Supplement: Supplementary file 1 [file oncotarget-08-67129-s001.pdf]

Remarkably similar CTLA-4 binding properties of therapeutic ipilimumab and tremelimumab antibodies

SUPPLEMENTARY MATERIALS

Supplementary Table 1: Sequences of ipilimumab-scFv and tremelimumab-scFv

Name	Sequence
ipilimumab-scFv	EIVLTQSPGTLSLSPGERATLSCRASQSVGSSYLAWYQQKPG<br>QAPRLLIYGAFSRATGIPDRFSGSGSGTDFTLTISRLEPEDF<br>AVYYCQQYGSSPWTFGQGTKVEIKR*GGGGSGGGGSGGGGSGG*<br>*GGS*QVQLVESGGGVVQPGRSLRLSCAASGFTFSSYTMHWVRQ<br>APGKGLEWVTFISYDGNNKYYADSVKGRFTISRDNSKNTLYL<br>QMNSLRAEDTAIYYCARTGWLGPFDYWGQGTLVTVSS
tremelimumab-scFv	DIQMTQSPSSLSASVGDRVTITCRASQSINSYLDWYQQKPGK<br>APKLLIYAASSLQSGVPSRFSGSGSGTDFTLTISSLQPEDFA<br>TYYCQQYYSTPFTFGPGTKVEIKR*GGGGSGGGGSGGGGSGGG*<br>*GS*QVQLVESGGGVVQPGRSLRLSCAASGFTFSSYGMHWVRQA<br>PGKGLEWVAVIWYDGSNKYYADSVKGRFTISRDNSKNTLYLQ<br>MNSLRAEDTAVYYCARDPRGATLYYYYYGMDVWGQGTTVTVSS
